# The Pivotal Distinction between Antagonists’ and Agonists’ Binding into Dopamine D4 Receptor—MD and FMO/PIEDA Studies

**DOI:** 10.3390/ijms25020746

**Published:** 2024-01-06

**Authors:** Paweł Śliwa, Magdalena Dziurzyńska, Rafał Kurczab, Katarzyna Kucwaj-Brysz

**Affiliations:** 1 Faculty of Chemical Engineering and Technology, Cracow University of Technology, Warszawska 24, 31-155 Kraków, Poland; 2 Department of Medicinal Chemistry, Maj Institute of Pharmacology, Polish Academy of Sciences, Smętna 12, 31-343 Kraków, Poland; katarzyna.kucwaj@uj.edu.pl; 3 Department of Technology and Biotechnology of Drugs, Faculty of Pharmacy, Jagiellonian University Medical College, Medyczna 9, 30-688 Kraków, Poland

**Keywords:** molecular dynamics, fragment molecular orbital, pair interaction energy decomposition analysis, dopamine D4 receptor

## Abstract

The dopamine D4 receptor (D4R) is a promising therapeutic target in widespread diseases, and the search for novel agonists and antagonists appears to be clinically relevant. The mechanism of binding to the receptor (R) for antagonists and agonists varies. In the present study, we conducted an in-depth computational study, teasing out key similarities and differences in binding modes, complex dynamics, and binding energies for D4R agonists and antagonists. The dynamic network method was applied to investigate the communication paths between the ligand (L) and G-protein binding site (GBS) of human D4R. Finally, the fragment molecular orbitals with pair interaction energy decomposition analysis (FMO/PIEDA) scheme was used to estimate the binding energies of L–R complexes. We found that a strong salt bridge with D3.32 initiates the inhibition of the dopamine D4 receptor. This interaction also occurs in the binding of agonists, but the change in the receptor conformation to the active state starts with interaction with cysteine C3.36. Such a mechanism may arise in the case of agonists unable to form a hydrogen bond with the serine S5.46, considered, so far, to be crucial in the activation of GPCRs. The energy calculations using the FMO/PIEDA method indicate that antagonists show higher residue occupancy of the receptor binding site than agonists, suggesting they could form relatively more stable complexes. Additionally, antagonists were characterized by repulsive interactions with S5.46 distinguishing them from agonists.

## 1. Introduction

**Figure 1:**
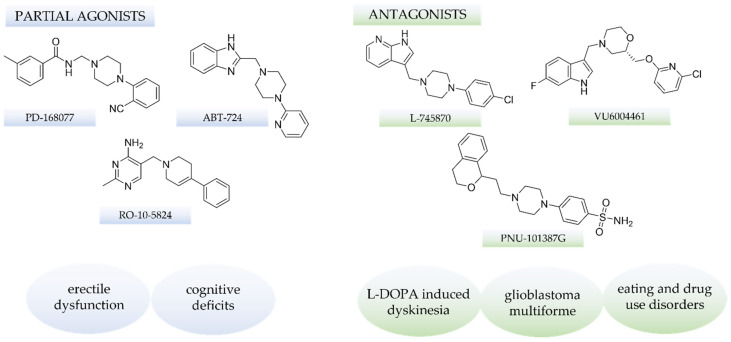
The exemplary structures of selective D4R ligands and the potential therapeutic significance of both antagonism and agonism confirmed in preclinical studies.

Attention to dopamine signaling was first focused over 60 years ago when chlorpromazine was serendipitously discovered as the first effective treatment for schizophrenia patients [1,2]. More than 20 years later, the target of early antipsychotic drugs was identified and validated. Nevertheless, it is reported now that the dopaminergic system consists of two subfamilies, D1-like receptors (D1R and D5R) and D2-like receptors (D2R, D3R, and D4R) [3,4], which remain the most known and explored dopamine receptors as the key antipsychotic targets. Its antagonists are widely used in patients with schizophrenia (first and second generation) and also for nausea and vomiting [5]. Notably, some of these compounds also have high (or even higher) affinity [6] to D4R, and this fact initially gave hope to omit the extrapyramidal effect of antipsychotics resulting from interaction with D2R via the use of selective D4R antagonists [7]. Unfortunately, clinical trials performed with such compounds confirmed no benefits for schizophrenia patients [8,9], which led to a further underestimation of this receptor as a therapeutic target. Thus, it remained in the shadow of D2R for many years. Interestingly, the situation has started to change, and now, many lines of evidence show the critical role of the D4R in non-schizophrenia pathophysiological processes. The exciting result comes from recent studies showing that selective D4R antagonists inhibit the growth of glioblastoma multiforme (GBM) neural stem cells (without cytotoxic effect on ‘normal’ neural stem cells and non-specific cytotoxic effect) [10]. Moreover, Dolma et al. confirmed that tested D4R antagonists are synergistic with temozolomide (the gold standard in GBM therapy), impairing the autophagy–lysosomal degradation pathway. This effect is accompanied by G0/G1 cell-cycle arrest and subsequent apoptosis. The potential practical application of such compounds was confirmed in GBM xenograft growth in vivo experiments [10]. Finally, the investigation of D4R expression in GBM patient-derived cell lines and analysis of The Cancer Genome Atlas (TCGA) led to the conclusion that patients with tumors with high D4R expression have worse survival rates than those with low D4R expression [10]. Moreover, studies with the same compound (L-745870, Figure 1) and structurally different D4R antagonist (VU6004461, Figure 1) indicate the ability to attenuate L-DOPA-induced dyskinesia (LID) [11,12]. Another study concerning the pharmacological role of D4R antagonism highlights its association with drug abuse and food addiction [13]. In contrast to disappointment with selective D4R antagonists in clinical trials in schizophrenia patients, a preclinical study in mice with D4R partial agonist (RO-10-5824, Figure 1) [14] and with PD-168077 (Figure 1) alone and with lurasidone showed the D4R agonism as a strategy to reverse the cognitive deficit in novel object recognition tests, thus, suggesting the potential for the treatment of schizophrenia-associated cognitive impairments [15]. Moreover, the importance of D4R’s role in erectile dysfunction has been very well reported. Several studies using D4R agonists (e.g., ABT-724, Figure 1) confirmed that they induce penile erection in animal models [16,17].

All these facts underline the returning interests of D4R as a promising therapeutic target in widespread diseases, and searching for both novel agonists and antagonists seems clinically relevant. To rationalize the design of novel compounds, the analysis of protein–ligand complexes is instrumental in defining the structural fragments that may be crucial to forming key interactions. Significantly, the binding mechanism to the receptor for antagonists and agonists differ. Thus, we performed profound computational studies in this work, highlighting the key similarities and differences in binding modes. Molecular dynamics (MD) simulations were performed to characterize the conformational dynamics of the D4R’s characteristic structural motifs [18,19,20] upon known agonists or antagonists binding. The dynamical network methodology [21,22] was also applied to explore the communication paths between the ligand and G-protein binding site of the D4R. Finally, the quantum fragment molecular orbitals method with a pair interaction energy decomposition analysis scheme (FMO/PIEDA) [23,24,25] was employed to estimate the differences in the binding energies between active and inactive states of ligand–receptor (L–R) complexes. The availability of crystal structure certainly increases the rationalization of implementation of the forced-field-based methods (such as MD); however, some types of molecular interactions are not adequately parametrized [23]. Therefore, to understand their high complexity, the additional application of full quantum mechanical calculations seems very useful. In FMO/PIEDA biomolecules, such proteins or nucleic acids are divided into substructures (fragments) and pair interaction energies (PIE) are calculated for such resulting individual fragments, making calculations more accurate than for whole large molecules [24,25,26]. The computational scheme proposed in this paper has been successfully applied previously to theoretical considerations of GPCR-targeted new drug design [27,28,29,30,31] as well as to deep insight into structure–activity relationship (SAR) analysis [32,33]. However, such a computationally comprehensive approach presented within this study has not been employed yet for D4R and the design of its ligands. The structural differences between antagonists and agonists, which determine their functional profile, have also not been characterized well in the literature; thus, the design of compounds with desired functional profiles remains challenging. Therefore, this work aimed to identify pivotal D4R–ligand interactions, which determine both D4R compounds’ affinity and also intrinsic activity.

## 2. Results and Discussion

**Figure 2:**
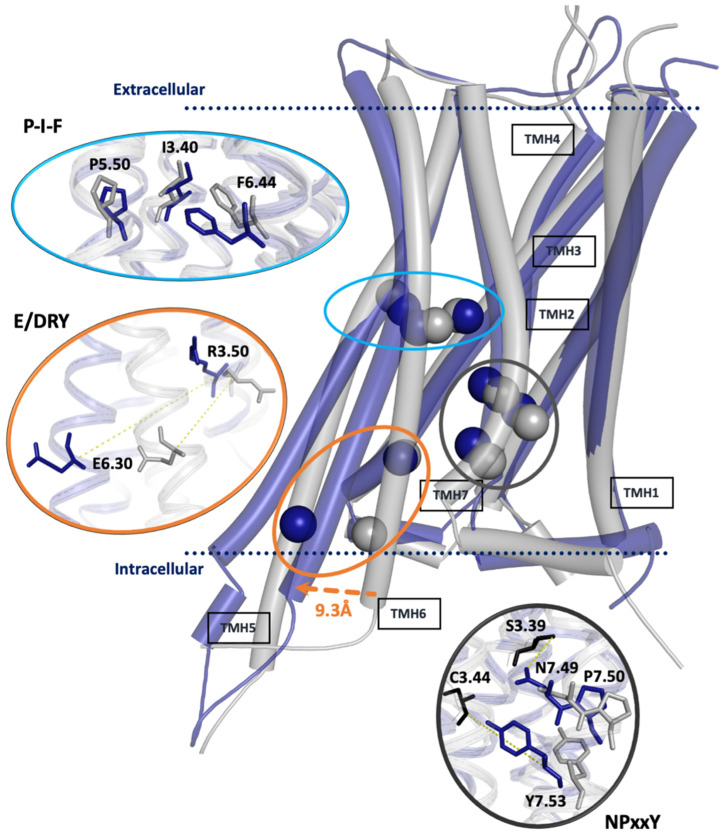
Superimposition of the active (purple–blue, homology model built on D2R template, PDBID: 6VMS) and inactive (grey, PDBID: 5WIU) structures of the human D4 receptor. Marked characteristic amino acid sequence motifs (P-I-F, E/DRY and NPxxY) of which the corresponding conformation occurs depending on the activation state of the receptor. The distinctive amino acids were provided in The Ballesteros–Weinstein numbering scheme, and the numbering of transmembrane helices (TMH1-7) was indicated.

The ability of GPCRs to undergo conformational changes is their primary ability related to signal transduction across the cell membrane. This mechanism is quite complex. The cascade of structural changes that allow G-proteins and other signaling proteins to bind to the intracellular surface of GPCRs is initiated by the binding of extracellular ligands, i.e., small hormones and neurotransmitters, but also peptides/proteins. In recent years, there have been significant advances in our knowledge of the flexibility and dynamics of GPCRs obtained through crystallography, spectroscopy, and computer simulations. Of all the conformational changes that GPCRs undergo, several appear to be barely crucial from a functional point of view. Reorganizations of transmembrane helices, especially TMH 5–7, play a key role in signal transfer across the membrane. Studies suggest that the transmembrane helices of GPCRs can adopt many different conformational states, not just one active and one inactive state. These states have different implications for receptor signaling. Typically, the largest conformational changes occur on the intracellular side of the GPCR, but the extracellular half of the receptor also changes conformation. In particular, the ligand-binding pocket undergoes subtle but essential structural changes [19]. The structural differences are summarized in Figure 2, where areas considered crucial in the activation process of most GPCR receptors are highlighted. However, there are some differences in the mode of activation among the GPCRs studied to date [19]. Agonist interactions in the binding pocket vary for different GPCRs, probably related to their evolved specificity/selectivity.

In recent years, there have been many advances in understanding the general structural, biochemical, and functional properties of dopamine receptors, which have led to the development of many pharmacologically active compounds that directly target dopamine receptors, such as drugs against Parkinson’s disease and antipsychotics [20,34,35,36,37]. Recent advances in the understanding of the complex biology of the activation mechanisms of other GPCRs provided the motivation for us to investigate and discuss the proposed activation mechanism for the human dopamine receptor D4, for which there is a high-resolution crystal structure of the inactive form (PDBID: 5WIU) [37] and a crystal of a close homologue in the active state (D2R, PDBID:6VMS) [38].

First, a database of compounds with agonists and antagonists towards the human D4 receptor was constructed. Activity data for 5997 compounds against the human dopamine D4R were found in the ChEMBL database. Among this set, 77 compounds showing agonist or partial agonist activity and 25 antagonists were extracted. These two subsets were then clustered due to their structural similarity, and representative molecules were identified (see Experimental). Finally, seven agonists (Figure S1) and ten antagonists of the human D4R (Figure S2) were selected for further studies. For this collection of compounds, molecular docking, molecular dynamic simulations, and stabilization energy calculations using the quantum chemical approach FMO/PIEDA were performed in subsequent steps.

### 2.1. Docking

A set of agonists and antagonists were docked to the active and inactive forms of D4R, respectively, using the IFD algorithm. This approach has been applied in other works [18,20,39]. The IFD docking algorithm only allows movement of the amino acid side chains in the immediate surroundings of the ligand for best fitting. For example, this can be seen in the slight differences in the side chain alignment of the C3.36 residue (Figure S3) for agonists. Unfortunately, docking did not allow the observation of any significant differences in the place and manner of binding of agonists relative to antagonists. As the ligand–protein complex is a dynamic system, the docking procedure is only partially suitable for studying the significance of individual interactions in receptor activation. However, based on a visual assessment of the molecules’ alignment in the binding pocket and the ability to form, characteristic of all aminergic receptors, a salt bridge with the aspartic acid residue (D3.32) [40], and taking into account scoring, the poses with the highest affinity, and the preferred mode of ligand binding in the complex, were selected (Figures S3 and S4). The chosen ligand–receptor complexes were applied sequentially as starting structures for molecular dynamics simulations.

### 2.2. MD Simulations

The activation of a GPCR protein upon ligand binding is a dynamic process [19]. It involves a specific rearrangement in its structure, which results in the transduction of extracellular signals into the cell. Therefore, it was reasonable in the next step to perform molecular dynamics simulations. From a methodological point of view, the time of the simulation may be of the most significant importance here. Over the past dozen years, the rapid development of computer resources has led to several attempts to run very long simulations above microseconds. The large-scale molecular dynamics simulations of adrenergic GPCRs [41,42] indicate that activation events often occur independently in short periods. However, a “fully activated” conformational state occurs only stably when a nanobody (i.e., G-protein) is bound [43]. After a sufficiently long period, the active-like complex of b-adrenergic receptor with agonist spontaneously changed to the inactive form in almost half of the simulations. The remaining complexes mainly adopt an intermediate conformation, distinguished from the active state only by the different orientations of TMH7 [41]. Most importantly, however, this relaxation is time-consuming. These above studies, as well as the most recent work [18], clearly indicate that for the first 35–100 ns, stabilization of the complex in the initial active form is observed. In addition, the authors have partially proven that the ligand could invoke the transient activation observed in the 5-HT1A/VXT MD simulation [18]. Therefore, in this work, 100 ns MD simulations were performed because the adopted model of the active D4R receptor was without an intracellular protein. The basic assumption of this approach is that the methodology used will allow for the observation of differences in the geometries and interaction energies in the complexes depending on the different functionalities of the ligands.

The stability of the simulated complexes was evaluated by examining the RMSF and RMSD profiles (Figure S5). Generally, both types of complexes are very stable throughout the simulation. The receptor in the inactive state underwent fewer changes when bound to the antagonist than the active one bound to the agonist. The reason was the increased mobility of ICL3 in the latter. Both types of ligands have evolved slightly during the simulation. These are caused by the tight binding site cavity in the D4R receptor and the very stable anchoring of the ligands by residues characteristic of all GPCRs (D3.32, aromatic complex in TMH6). A comparison of docking complexes with representative structures from MD is shown in Figures S3 and S4.

The following two subsections analyze the differences in the geometry of D4R complexes with agonists and antagonists and report the results of the dynamical network analysis.

#### 2.2.1. Influence of Agonists/Antagonists on Receptor Dynamics

The main determinants of structural changes in GPCR receptors are specific sequence motifs indicating characteristic sites involved in the most significant conformational changes of the receptor as a result of activation. Critical areas in class A GPCRs, including the D4R receptor, involve the ligand-binding site (LBS), P-I-F, NPxxY, and E/DRY motifs, which play a key role in conformational stabilization (Figure 2) [19].

**Figure 3:**
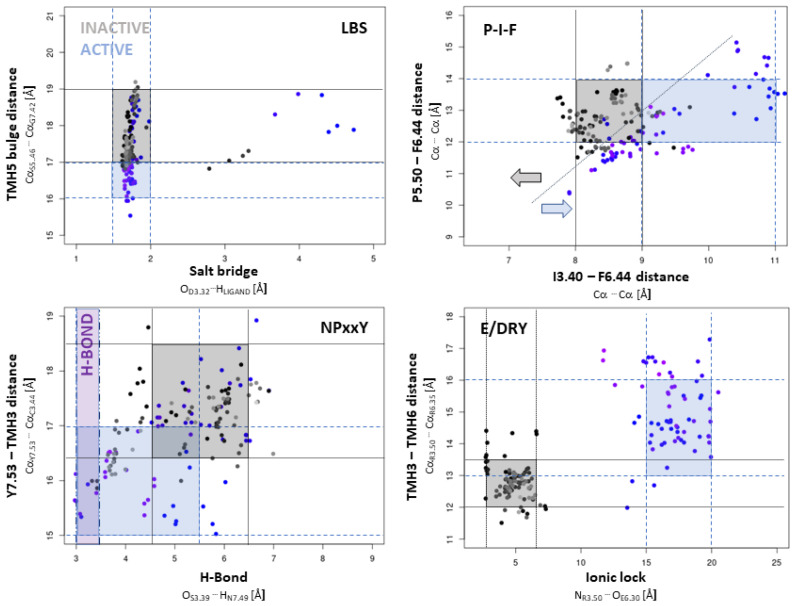
The multivariate plot shows measured geometric parameters’ changes of characteristic structural motifs related to receptor activation. The points mean the medians of the representative distances from 10 ns sections of each trajectory. Shades of purple indicate points of agonists, and shades of grey for antagonists complexes. The shaded areas correspond to regions characteristic of a specific receptor activation state, with gray indicating the inactive state and violet representing the active state.

Structural changes within the ligand-binding site appear to be the most subtle in GPCRs [19] and seem impossible to catch in virtual screening experiments. Observations of crystal structures indicate a minimal tightening of the binding cavity in the case of active conformation [44,45]. In this paper, following Ladefoged et al. [18], we measured it as the distance between Cα atoms of residues 5.46 and 7.42. Based on the data from the 100 ns MD simulation (Figures S6 and S7), a multivariate plot was prepared (Figure 3). In the case of the D4R–agonists complexes, we observed such tightening of the binding site, however, it persisted for a short time. The reason may be the inability to form, for most studied agonists, a hydrogen bond with S5.46, observed for the BI-167107 in the β2-adrenergic receptor [45], and dopamine in the D4 receptor [20].

The NPxxY motif is located at the G-protein binding site at the intracellular end of helix 7 (TMH7). A distinctive feature of receptor–agonist complexes is the conformation of tyrosine Y7.53 and aspartate N7.49 (Figure 2). This bulge of the side chains allows the separation of TMH6 from TMH3 and the formation of attachment points for the corresponding amino acids of G-protein. MD studies for β2AR have shown that during receptor inactivation, the binding site of G-protein usually adopts an intermediate conformation different from both active and inactive crystallographic ones. After transitioning to this intermediate, TMH7 assumes an inactive conformation, and its intracellular end moves away from helices 3 and 6 [41]. In this work, structural changes concerning this motif are shown in Figures S6 and S7 as differences in the distances of serine S3.39 to asparagine N7.49 (O^…^H distance marked as an orange line) and cysteine C3.44 to tyrosine Y7.53 (Ca^…^Ca distance marked as a black line) during the simulation. This first amino acid pair can form a hydrogen bond under favorable conditions. No significant differences are observed between the active and inactive structures. Still, in the case of antagonist binding, the distance between the amino acids slowly changes compared to the complex with the agonist. The distance between the donor and acceptor of a potential hydrogen bond is about 5 Å, which means no binding. In the second case, i.e., Y7.53^…^C3.44, the distances are significantly different between the two studied types in the initial phase of the simulation. It can be seen that the binding of the agonist initially causes a decrease in the distances, but by the middle of the simulation, the distances are almost equal. Figure 3 shows the above data for all simulations in a multivariate plot, where the areas characteristic of the complex type are marked. It can be seen that the distinct distances for the inactive conformation (grey points) mostly fall within the marked area, in contrast to the active-like one. These results indicate that the simulated D4R complexes with the agonist probably adopt an intermediate conformation capable of G-protein attachment.

The P-I-F motif, the so-called “switch of TMH 6 and 5”, is located at the point of inflexion of these helices (Figure 2). In the inactive state, helices 3, 5, and 6 in the intracellular part are aligned approximately parallel. Contrary to the above, in the active state, from this point, there is a twisting of TMH3, characteristic of all GPCRs, and, more importantly, a deviation of helices 5 and 6 from the vertical axis of the receptor. The study on the b2-adrenergic receptor suggests that this amino acid motif is responsible for an allosteric communication between the ligand-binding site and the G-protein binding site [44]. According to Dror et al., the mechanism proceeds as S5.46 and P5.50 move into the space occupied by I3.40 in the inactive structure. I3.40 consequently moves towards TMH6 into the space previously occupied by F6.44, which moves away from TMH3, deflecting the intracellular end of TMH6 away from TMH3 [41]. The dopamine D4 receptor activation study has also suggested that the serine complex in TMH5 (S5.42, S5.43, S5.46) acts as an H-bond switch causing receptor activation [20]. Herein, the dynamic of this motif was monitored by measuring the distance between the Ca atoms of P5.50, I3.40, and P6.44 (Figures S6 and S7), as in the publication of Ladefoged et al. [18]. The overall results (Figure 3, P-I-F) show that for antagonists, again, the characteristic distances are within the experimental range. For agonists, we observe events where helices 6 and 3 are separated by an assumed distance only in the initial few tens of nanoseconds. Generally, a good approximation is that the points for the two types of complexes tend to align on the two sides of the drawn diagonal. The mechanism of the D4R activation observed here, as a result of agonist binding, appears to involve the approximation of helix axes 6 and 3 at this point, with the consequent effect of moving the intracellular ends of helices 6 and 5 away from the vertical axis of the receptor (like a fracture).

The most visible effect of transforming the receptor to the active form is the deflection of the ends of intracellular helices 5 and 6. Hence, spectacular differences are observed between the distances between the amino acids in the so-called E-DRY motif that characterizes this effect. This motif is located on helices 3 and 6 close to the intracellular part (Figure 2). Upon activation, the distance at this site between TMH3 and TMH6 increases significantly (about 4 Å), as illustrated by the results obtained in this work (Figure 3, Figures S6 and S7). The distances between the side chains of the amino acids arginine R3.50 and glutamic acid E6.30 in the active structure are considerably more prominent than in the inactive form and reach nearly 20 Å. In the inactive form of the receptor, these two residues could form an ionic bond, which further stabilizes this conformation. In both cases, the measured distances remained nearly fixed throughout the simulation, so both types of complexes at this location can be considered stable. These structural changes form a cavity on the inside of the receptor, which allows binding to the G-protein.

#### 2.2.2. Signal Transfer Based on the Dynamical Network Analysis

The dynamical network analysis (DNA) makes it possible to predict how certain areas of a protein can potentially ‘communicate’. The pathway of interactions between distinct protein parts can be determined [21,22]. To deeper analyze the mechanism of activation of the human dopamine D4 receptor, the following DNA studies of the possibility of signal transduction from the ligand to the end of helix 6 in the receptor, namely, the R6.29 residue located in the intracellular part, for both types of intrinsic ligand activity were performed. This is one hypothesis for the mechanism of GPCR receptor activation, assuming allosteric communication between the ligand binding site (LBS) and the G-protein binding site GBS occurs by nonbonded interactions between residues in a protein [44]. In the DNA method, the probability of information transfer between two residues is based on the anti-correlation of the movement of the residues in question (Materials and Methods section). Each residue is mapped as a node, and the internode communication is denoted as an edge. The weight of an edge represents the strength of communication between the nodes and is determined based on the degree of anti-correlated motion between them [21,22].

Recently, the LBS and GBS communication, based on the DNA approach, was studied for serotonin receptors (5-HT1A, 5-HT1B, and 5-HT7) complexed with multimodal antidepressant vortioxetine [18]. There, the suboptimal communication pathways from D3.32, which anchors the agonist to the LBS, to E6.30 were determined. Only in the case of 5-HT1A-vortioxetine, with the communication directly via I3.40 and F6.44, from the P-I-F motif, was this observed. In the other repeat simulations, many alternative pathways were detected. The authors concluded that it meant the LBS was not directly involved in the communication pathway in the latter simulations [18]. The above approach ignores the importance of other ligands–residue edges, i.e., it is assumed that the signal must start at D3.32. A similar procedure, namely, MOdular NETwork analysis (MONETA), which employs the concept of communication propensity, has been previously utilized to study allosteric network pathways in unbounded WT and mutants, V194G and R237L, of human dopamine D4R. In the native protein, the inter-transmembrane contacts were intact, while in both mutant networks, they were lost [39].

**Figure 4:**
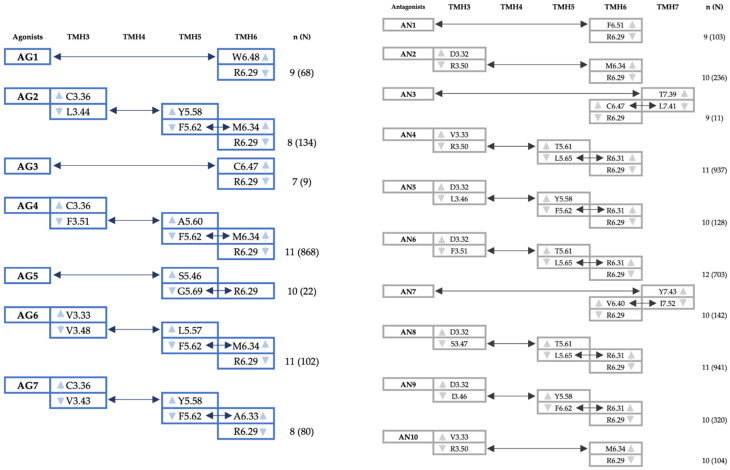
Dynamic network analysis of seven D4R/agonist (AG1–AG7) and D4R/antagonists (AN1–AN10) complexes. Optimal pathways between the ligand and R6.29, located at the end of TMH6, are shown. Each column represents the residues from a given helix, which are the jump points from one helix to the other. Light blue arrows indicate communication along a helix, while dark blue arrows indicate signal transfer between helices. n is the number of residues involved in a given optimal pathway, and N is the number of possible paths found.

For aminergic GPCRs, it has been assumed that the ligand anchors most often on aspartic acid D3.32 [40] and one would take that information transport should continue from this point. However, here, we have performed the analysis assuming that the signal initiates the ligand interaction with the residue with which the DNA approach most weights the contact. Examples of the communication networks between the ligand and TMH6 end are shown in Figure S8, separately for the agonist and antagonist. There are many possible connections of different lengths between the residues in question, but they are not equal. In the approach used here, the optimal pathway is the one with the amino acid nodes/residues occurring in the greatest number of pathways and guaranteeing the shortest communication in the complex. All optimal pathways are summarized in Figure 4 below. The number of suboptimal pathways (N) informs about the path degeneracy, and the distance of the path n (defined as the sum of the edge weights along a given path) tells about the allosteric strength.

In general, several interactions characteristic of agonists and antagonists can be observed when considering ligand–receptor interactions. In the case of the first group, we keep a tendency to form the most favorable communication pathways between the ligand and the binding pocket, in which the cysteine C3.36 residue binds directly to the agonist. In addition, the tryptophan W6.48, cysteine C6.47, serine S5.46, and valine V3.33 residues also turn out to be crucial in single networks. In the case of antagonists, the dynamic networks are clearly dominated by contact with aspartic acid D3.32. Furthermore, the possibility of initiating the investigated communication pathway was observed by interacting with residues from helices 6 and 7 (F6.51, T7.39, and Y7.43). These observations potentially offer another mechanism for allosteric communication between the agonist, especially those without the ability to hydrogen bond with S5.46, and the GBS involving C3.36 and perhaps T3.37. These residues are located in the immediate surroundings of I3.40 (Figures S3 and S4) and interact with it, enabling F6.44 rotation. This hypothesis needs confirmation whether it is only typical of the dopamine D4 receptor or may occur for other GPCRs.

### 2.3. The Importance of Repulsive or Attractive Forces in Activating D4R—FMO/PIEDA Calculations

**Figure 5:**
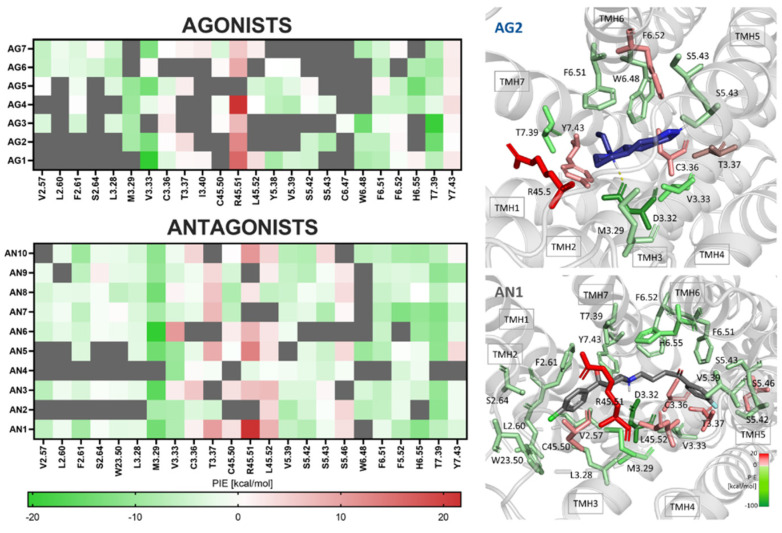
Energy array of ligand–receptor complex stabilization by individual amino acid residues at the receptor binding site that is pair interaction energies (PIE), estimated by FMO approach; red—repulsive effect, green—attractive effect, grey—no contact, i.e., the distance to the given residue was longer than 4.5 Å. The interactions with D3.32 have been omitted on the heat map. On the right panel is the dopamine D4 receptor ligand binding site occupied by an exemplary agonist (AG1) or antagonist (AN1). The residues involved in ligand binding are colored according to the interaction energy (PIE, kcal/mol), with the scheme that shades of green are stabilization and red are repulsions.

After MD simulations, the complex stabilization energies were calculated using the FMO/PIEDA method for representative L–R structures. The heat map shown in Figure 5 illustrates the critical amino acid residues for ligand–receptor interactions. Most interactions observed during ligand binding by the dopamine D4 receptor are attractive (green color in the graph), with a minority of interactions having a repulsive effect (red color in the chart). The results of the energy decomposition analysis (PIEDA, Figure S9) showing the nature of the individual contacts and comparison of the calculated FMO binding energies (E_bind_) and binding free energies (DG_bind_) with K_i_ are included in Supplementary Materials. The results are consistent with other work on GPCRs with structurally diverse molecules, where a weak correlation is observed between calculated values and the experimental inhibition constant [31,46]. However, the FMO approach has been shown to work very well in a structure-based drug design (SBDD) for non-receptor tyrosine kinase inhibitors [23] and ligand selectivity analysis of the 5-HT1A receptor [30].

The D4R binding site is relatively conserved among D2-like receptors. It consists of residues: D2.50, D3.32, V3.33, C3.36, V5.39, S5.42, S5.43, S5.46, W6.48, F6.51, F6.52, H6.55, T7.39, and Y7.43 [39]. An earlier study has proved that three serine residues in TMH5 (S5.42, S5.43, and S5.46) are crucial for the binding of dopamine and other phenolic agonists by forming H-bonds with hydroxyl groups [20]. All typical bonds were observed in the complexes considered in this study (Figure 5). A salt bridge, characteristic of D4 ligands, between the aspartate anion of D3.32 and the ligands’ protonated nitrogen was present for both agonists and antagonists. This bond is not shown on the heatmap in Figure 5 due to the energy value being too high (approximately −100 kcal/mol), which would make the other values difficult to decipher. It was noted that antagonists engage more amino acid residues in the D4 receptor binding site than agonists, suggesting that they generally form more stable complexes. The results of the FMO/EDA calculations also provide some insight into previously suggested activation mechanisms, such as the interactions mentioned above of agonists with the serine complex in TMH5. In the tested set of agonist complexes with D4R, only AG6 formed polar bonds with S5.43 (H-bond) and V5.39 (halogen bond) after docking. These were broken during MD and were no longer observed in the representative structure (Figure S3). Although potentially AG3 and AG7 could also form such interactions, however, both docking and MD indicate a different active alignment. Interestingly, none of the agonists interacted with S5.46, as the FMO/PIEDA analysis also showed. The most interesting, however, is the result for the antagonists, where there is clearly an interaction with the serine system, and fascinatingly with S5.46 was repulsive. This interaction may, therefore, be responsible for the mechanism of inhibition of the human D4R receptor. The observed results indicate a direction for designing chemically original ligands with not only potential high D4R affinity but also with a desired functional profile. However, further research among a larger group and experimental verification is needed.

## 3. Materials and Methods

### 3.1. Protein and Agonists/Antagonists Set Preparations

The 3D structure of the human dopamine D4 receptor was downloaded from the Protein Data Bank database [47] (PDB ID: 5WIU). It is a protein crystal in complex with the antagonist nemonapride [37]. After removing all co-crystallized small molecules, this structure served as a model for the inactive conformation of the receptor. In this work, the active conformation of the D4 receptor was used as a homology model built on a template of the active form of the D2 receptor with PDBID 6VMS. The pre-prepared one was downloaded courtesy of the GPCRdb [48,49,50,51] just before the 2023 update, where AlphaFold2 was used [52]. The agonists and antagonists of the human D4 receptor sets of compounds were constructed using affinity results for 5997 compounds found in the ChEMBL database [53,54]. From this set, 77 compounds showing agonist or partial agonist activity and 25 antagonists were extracted manually by verifying the results of the source studies. All compounds were hierarchically clustered using Molprint2D, the Tanimoto metric, and the complete cluster linking method implemented in Canvas from the Schrödinger [55,56]. The centroid was selected from each cluster containing more than two members to constitute representative structures to further study. Two sets of test compounds were created, enlisting seven agonists and ten antagonists of the human dopamine D4 receptor, respectively (Figures S1 and S2).

### 3.2. Docking

The ligand three-dimensional structures were prepared using LigPrep 3.7 [57]. The appropriate ionization states at pH = 7.4 were assigned using Epik [58]. The protein preparation wizard was used to give the bond orders and appropriate amino acid ionization states [59]. The docking was performed using the induced-fit docking (IFD) protocol from Schrödinger. This method combines flexible ligand docking using the glide algorithm with receptor structure prediction and side chain refinement in prime (refine residues within 5.0 Å of ligand poses). The extended sampling protocol was used, which generates up to 80 poses per ligand with automated docking. In each case, the centroid of the grid box was fixed on D3.32 and allowed residue refinement within 5 Å of the ligand poses. The selection of the complex for further modelling was based on the values of docking scores and the consistency of the generated pose with known binding mode.

### 3.3. Molecular Dynamic and Analysis

The all-atom 100 ns molecular dynamics simulations were performed using program NAMD 2.13 [60], with standard all-atom forcefield CHARMM36 [61,62]. Topology parameters were created in CGenFF 1.0.0 [61]. The membrane and simulation system was built with the QwikMD beta [63] tool in VMD 1.9.3 [64], using a membrane of POPC (1-palmitoyl-2-oleoylphosphatidylcholine) explicitly solved 0.15 mol/L aqueous NaCl. The simulation was prepared in the sequence: minimization (2000 of 2 fs steps, NpT, 0 °C, 1 atm, restrained backbone), annealing (144,000 of 2 fs steps = 288 ps, NpT, 27 °C, 1 atm, restrained backbone), equilibration (500,000 of 2 fs steps = 1 ns, NpT, 27 °C, 1 atm, restrained backbone), and MD (50,000,000 of 2fs steps = 100 ns, 27 °C, 1 atm).

The binding free energies as the molecular mechanics Poisson–Boltzmann surface area (MM/PBSA) were estimated using a VMD Plugin named calculation of free energy (CaFE) [65], where the gas-phase energy difference between the complex and the separated receptor and ligand is obtained by calling NAMD. Then, the polar solvation-free energy is calculated by numerically solving the PB equation implemented in APBS [66]. Subsequently, the difference in solvent-accessible surface area (SASA) is measured, and the non-polar solvation-free energy is estimated by its approximate linear relation with SASA. At last, the binding free energy is summed and averaged throughout an ensemble of conformations. The root mean square fluctuation (RMSF) during MD simulations was measured using the VMD Tlc script. The root mean square deviation (RMSD) of the C alpha atomic coordinates was calculated using a built-in tool of UCSF Chimera 1.16 [67]. Clustering of the MD trajectories to obtain a structure representative for FMO/PIEDA calculations was performed using the MDMovie tool implemented in UCSF Chimera 1.16.

The probability of information transfer, that is, allostery between two residues, is based on the (anti)correlation of movement of the residues in question. For each simulation, each Cα atom was mapped as a node, and non-neighboring nodes were connected by so-called edges. Edges were assigned a weight based on the magnitude of the calculated correlation in motion between the two nodes. (Sub)optimal communication pathways are calculated as the shortest distance between the two residues regarding edge weights [21,22]. The dynamical networks were visualized using NetworkView in VMD 1.9.3 [64]. The correlation of the motions of atoms was calculated using CARMA [68] and suboptimal paths were calculated using a script obtained from the Schulten group webpage [69]. Only protein and ligand heavy atoms were included in the calculation, and nodes were assigned to Cα atoms of residues and basic nitrogen of ligands. The correlations of the motion of atoms within the same residue and the nearest neighbor were excluded from the analysis. The suboptimal paths linking D3.32 to the most intracellular end of TM6 (R6.29 for D4) were calculated using an edge length offset of 20; that is, the length of the longest suboptimal path must be less than 20 longer than the optimal path.

### 3.4. FMO/PIEDA

To study the importance and the nature of L–R interactions, single-point FMO–EDA [25] calculations were performed for the all-optimized complexes at the MP2/6-31G* [70] level using the GAMESS software [71,72]. The FMO calculations were performed for the ligand and receptor binding site. The FMO input commands were set as default. The pair interaction energies (PIE) and all contributions to total energies (electrostatic—*E_es_*, dispersion—*E_dis_*, charge-transfer—*E_ct_*, exchange repulsion—*E_ex_*, and the Gibbs solvation energy—*DG_solv_*) were calculated as previously described [25]. The Gibbs solvation energy was calculated based on the PCM model. FACIO [73] was used to prepare the systems and analyze the results. To illustrate the nature of interactions with individual amino acid residues, the percentage share of the sum of the absolute values of electrostatic contribution and charge transfer concerning the sum of the absolute values of the three contributions *E_es_*, *E_ct_*, and *E_disp_*_,_ was defined according to the formula:

(1) %Ees+ct=Ees+EctEes+Ect+Edisp·100%

In this way, the value is always positive and considers the different signs of contributions. The above parameter could be read so that 100% means a purely polar interaction. The value 0% means simultaneously 100% of the dispersion energy, which could be interpreted as a strictly hydrophobic effect.

## 4. Conclusions

Based on the molecular modelling techniques (docking, molecular dynamics, quantum chemical calculations), we identified the key interactions for bonding of ligands with different functionality for human dopamine D4 receptors. This allowed us to speculate and verify the possible mechanism of activation and inhibition for this receptor. The agonist binding results in forming an active receptor conformation, where helices 5 and 6 show a significantly larger bending angle than in the case of an unbound or antagonist-binding one. When an antagonist is bound, the binding site of the G-protein is locked.

The dynamic network analysis illustrated how residues in the P-I-F motif could transfer the ligand-induced information to the G-protein binding site. This is another step towards a more complete understanding of the activation mechanism of the D4R and other GPCRs. In the case of antagonist binding, we found that forming a salt bridge with D3.32 residue plays the most significant role in stabilizing the inactive receptor conformation. In contrast, agonists tend to create favorable communication between the binding pockets by contact with the cysteine C3.36. We believe this was due to the inability to form a hydrogen bond with S5.46 in the tested set of compounds, previously indicated as crucial.

The energy calculations using the FMO/PIEDA method indicate that most of the interactions observed during ligand binding by the dopamine D4 receptor were attractive. Antagonists showed higher residue occupancy of the receptor binding site than agonists, suggesting they could form relatively more stable complexes. Additionally, antagonists were characterized by repulsive interactions with S5.46, distinguishing them from agonists.

The results will enable the pharmacological classification of new compounds, which may help rationalize new functional D4 receptor ligands with optimized and more effective pharmacological properties.

## Data Availability

The input data and parameters are available for download at https://mckpk-my.sharepoint.com/:f:/g/personal/pawel_sliwa_admin_pk_edu_pl/EmzzDN_Re09Ai9cLZnJ42F4BIiYf9vkPJ7o5uO0IapLXiA?e=7zoeAm, accessed on 1 January 2024.

## Supplementary Materials

The following supporting information can be downloaded at: https://www.mdpi.com/article/10.3390/ijms25020746/s1.
