# Supplementary material for: The Pivotal Distinction between Antagonists’ and Agonists’ Binding into Dopamine D4 Receptor—MD and FMO/PIEDA Studies
Source: Int J Mol Sci. 2024 Jan 6;25(2):746. doi: 10.3390/ijms25020746 (PMC10815553; doi:10.3390/ijms25020746)

Supplementary materials

The pivotal distinction between antagonists' and agonists' binding into dopamine D4 receptor – MD and FMO/PIEDA studies

Paweł Śliwa [1,2,] *, Magdalena Dziurzyńska [1], Rafał Kurczab [2] and Katarzyna Kucwaj-Brysz [2,3]

[1] Faculty of Chemical Engineering and Technology, Cracow University of Technology, Warszawska 24, 31-155 Kraków, Poland
[2] Department of Medicinal Chemistry, Maj Institute of Pharmacology, Polish Academy of Sciences, Smetna 12, 31-343 Cracow, Poland; kurczab@if-pan.krakow.pl
[3] Department of Technology and Biotechnology of Drugs, Faculty of Pharmacy, Jagiellonian University Medical College, Medyczna 9, 30-688 Kraków, Poland; katarzyna.kucwaj@uj.edu.pl
* Correspondence: pawel.sliwa@pk.edu.pl; Tel.: +48-126282758

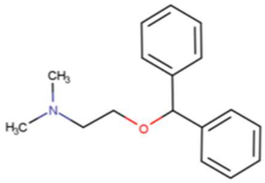

AG1

K_i = nd

DIPHENHYDRAMINE

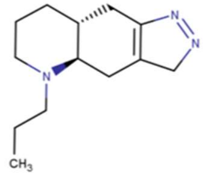

AG2

K_i = 8.50 nM

QUINPIROLE

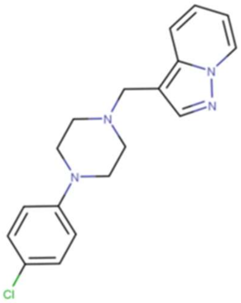

AG3

K_i = 3.60 nM

FAUC113

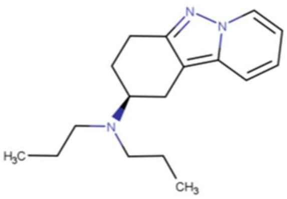

AG4

K_i = 50 nM

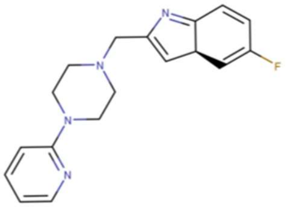

AG5

K_i = 3.60 nM

CP226269

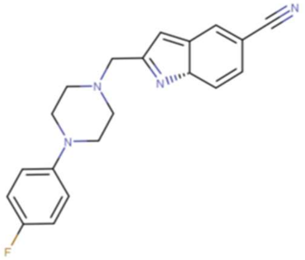

AG6

K_i = 1.0 nM

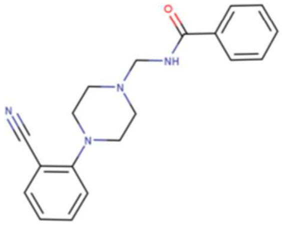

AG7

K_i = 27.0 nM

Figure S1. The chemical structures of the human **D4 receptor agonists** used in the study. Ki inhibition constant values based on data from the ChEMBL database.

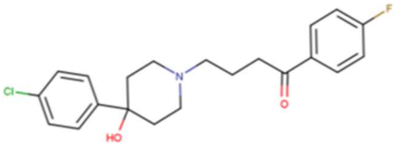

AN1
K_i = 12.7 nM
HALOPERIDOL

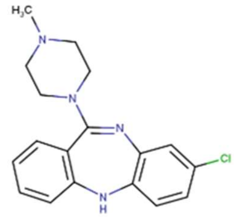

AN2
K_i = 16.0 nM
CLOZAPINE

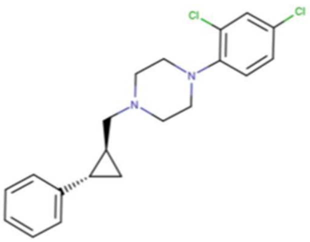

AN3
K_i = 24.0 nM

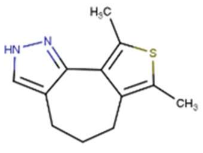

AN4
K_i = 12.0 nM

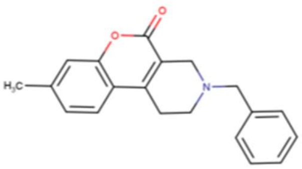

AN5
K_i = 3.6 nM

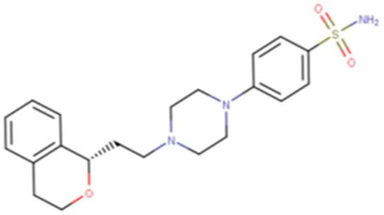

AN6
K_i = 6.8 nM
SONEPIPRAZOLE

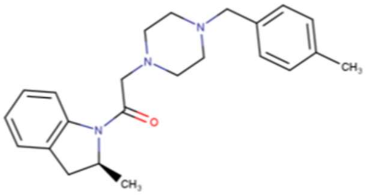

AN7
K_i = 7.0 nM

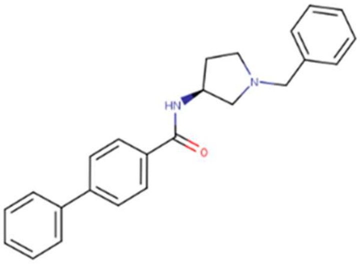

AN8
K_i = 3.0 nM

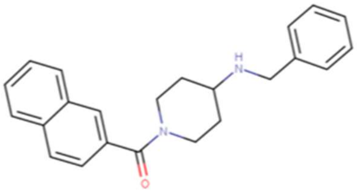

AN9
K_i = 167.0 nM

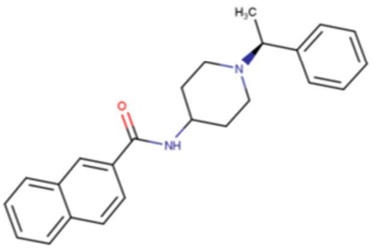

AN10
K_i = 71.0 nM

Figure S2. The chemical structures of the human **D4 receptor antagonists** used in the study. Ki inhibition constant values based on data from the ChEMBL database.

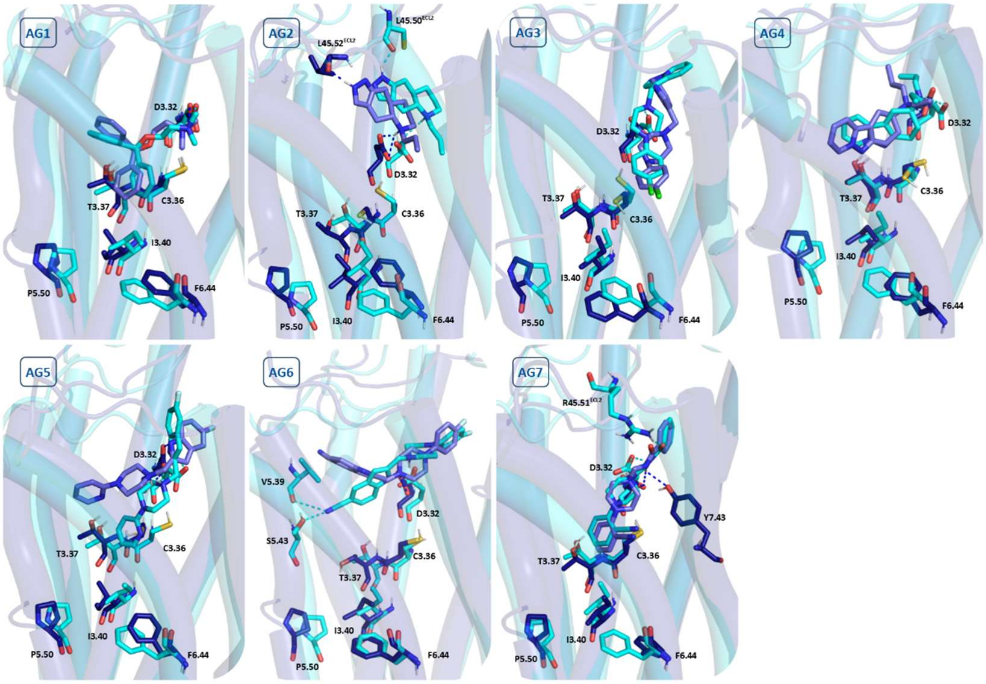

Figure S3. Superimposition of conformation from IFD (cyan) with representative structure after trajectory clustering from 100ns MD simulation (blue) of **agonist** complexes with human D4 receptor.

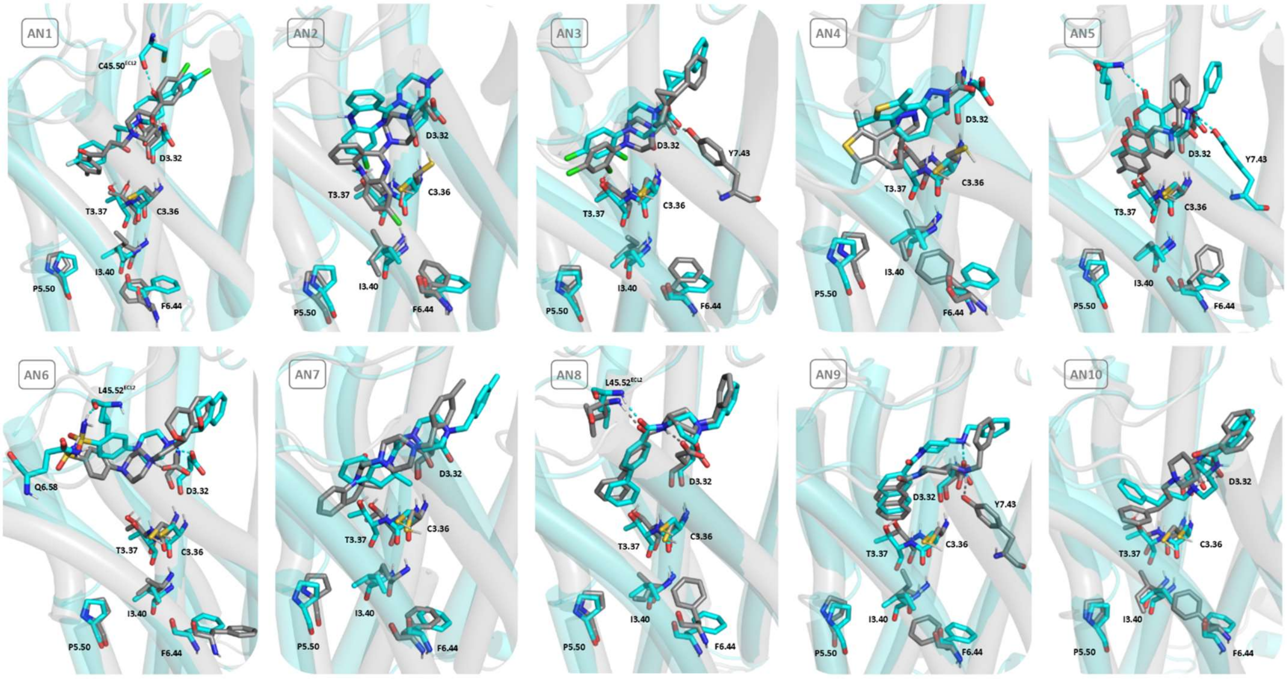

Figure S4. Superimposition of conformation from IFD (cyan) with representative structure after trajectory clustering from 100ns MD simulation (grey) of **antagonists** complexes with human D4 receptor.

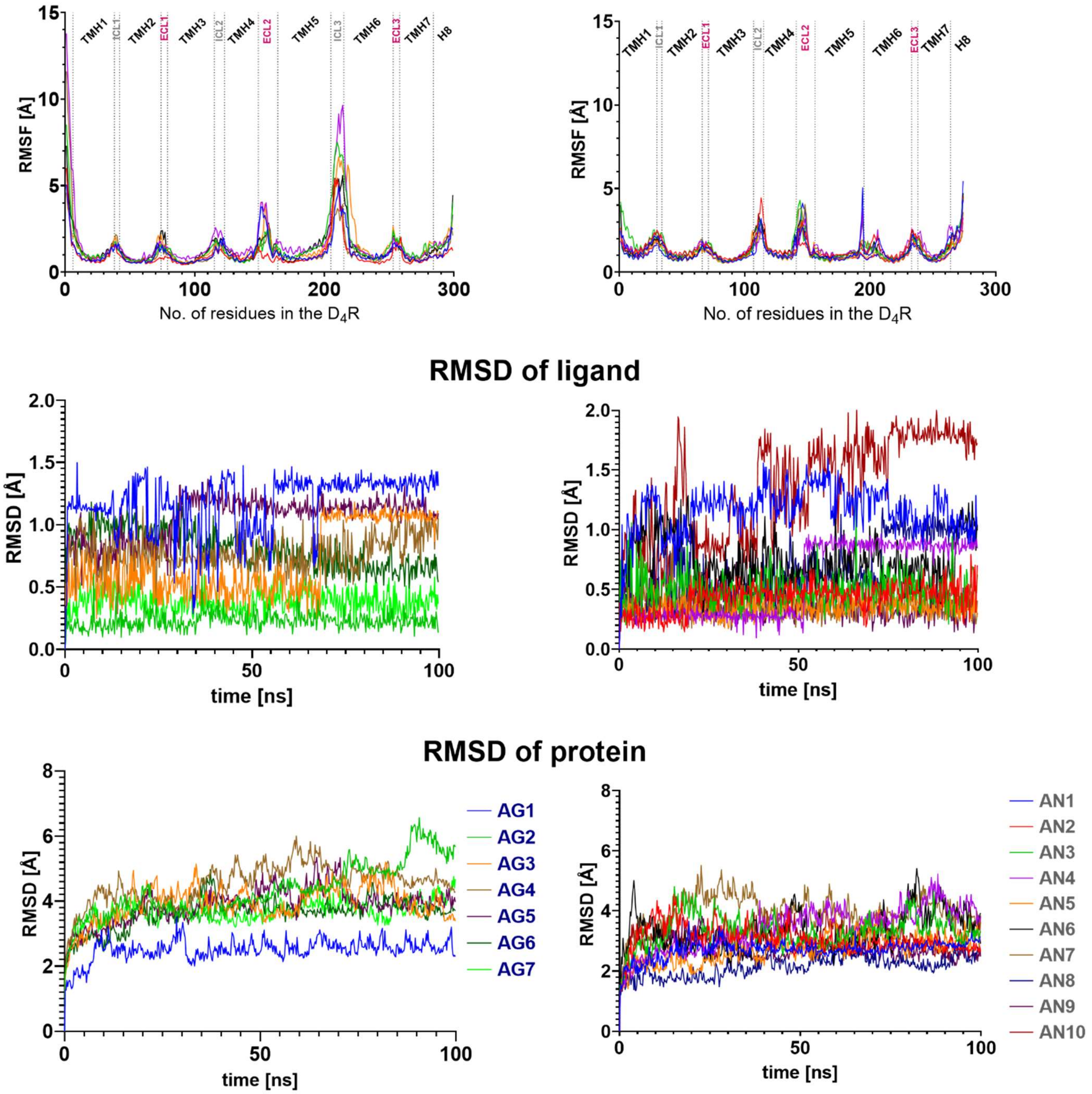

Figure S5. RMSF and RMSD during the 100 ns simulations for the complexes of agonists (AG1-AG7) and antagonists (AN1-AN10) with human dopamine D4 receptor.

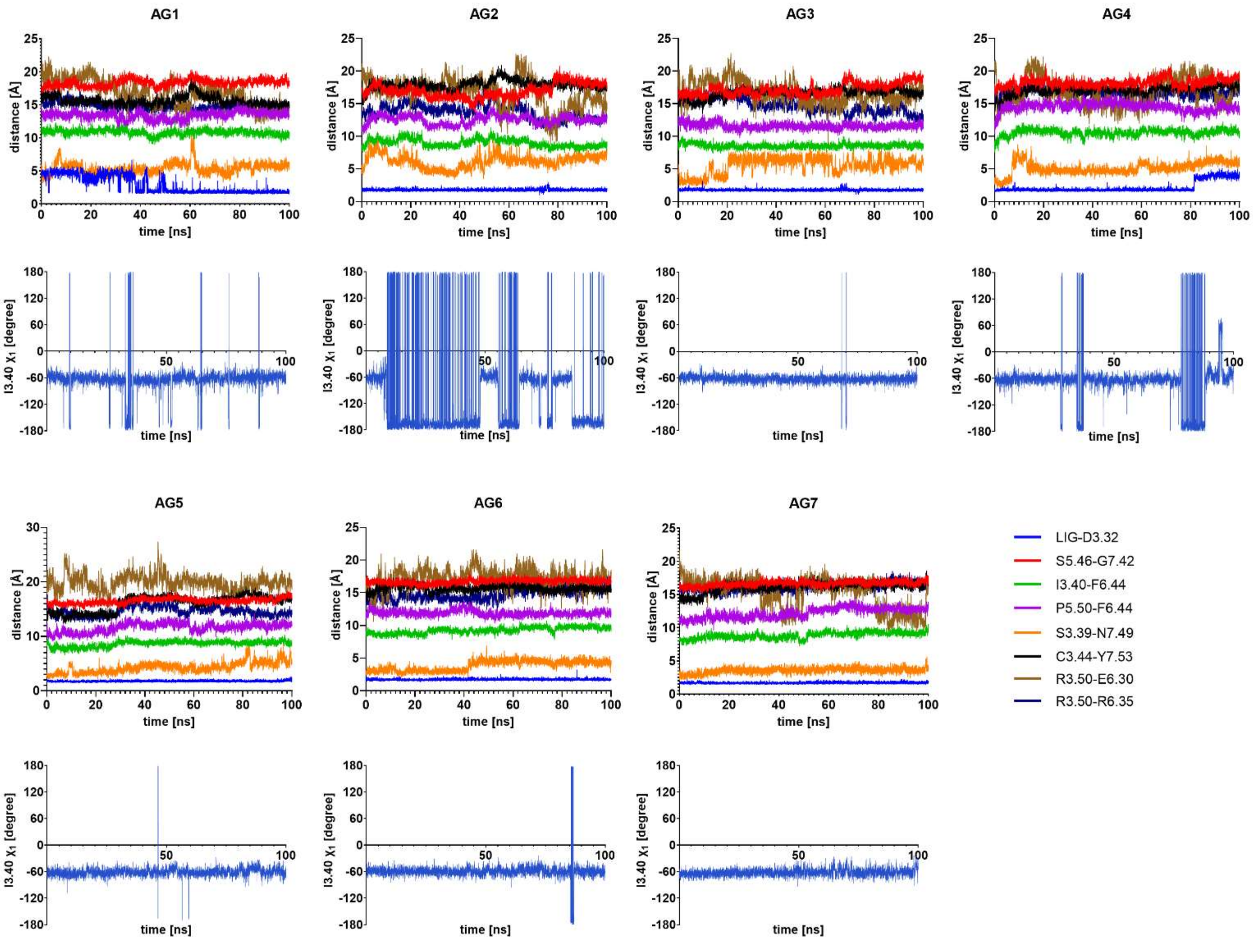

Figure S6. Measures of receptor activation/inactivation events based on changes in characteristic motifs throughout 100ns MD simulation of D4 receptor complexes with agonists. The time-progressed degree or distance changes are shown for (LIG-D3.32 and S5.46-G7.52) LBS, (I3.40-F6.44, P5.50-F6.44 and I3.40 χ_1) P-I-F, (S3.39-N7.49 and C3.44-Y7.53) NPxxY, (R3.50-E6.30) ionic lock, and (R3.50-R6.35) TMH3-TMH6 distance.

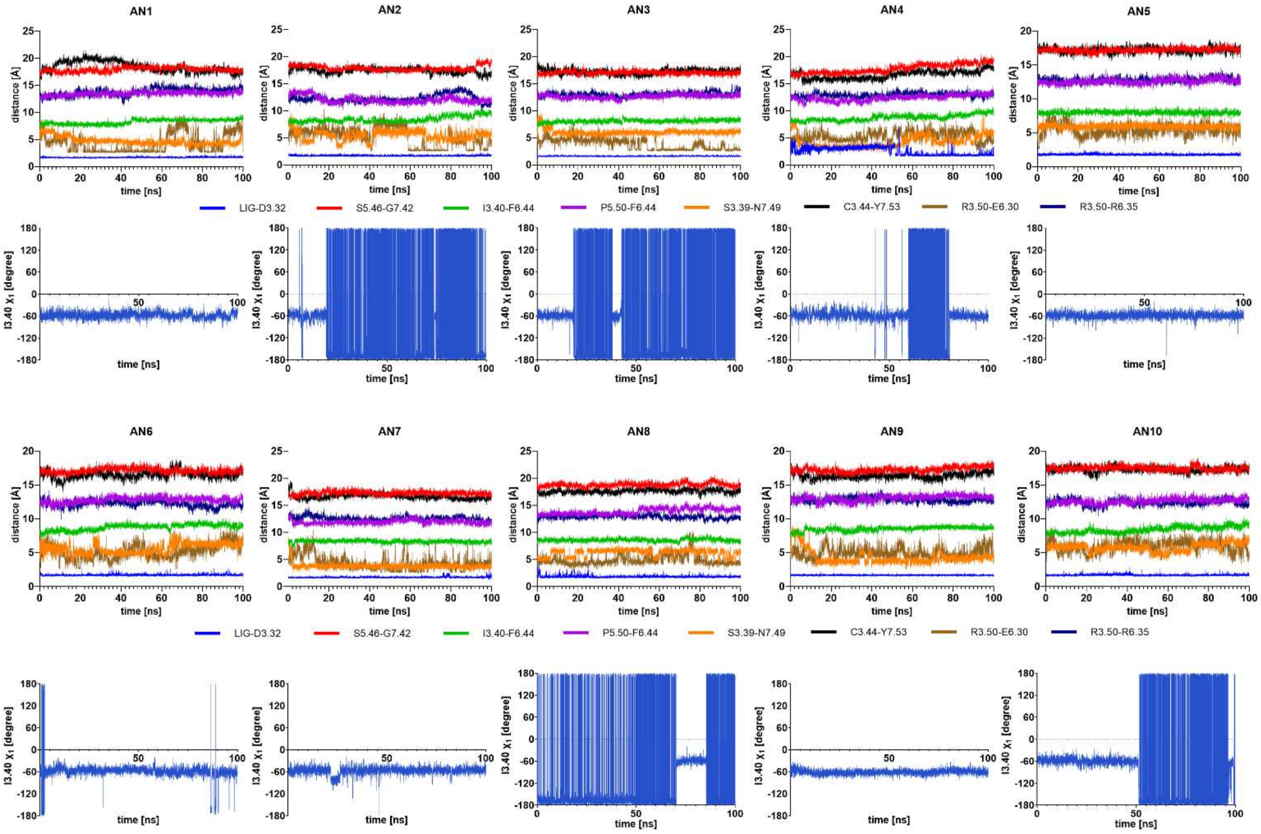

Figure S7. Measures of receptor activation/inactivation events based on changes in characteristic motifs throughout 100ns MD simulation of D4 receptor complexes with antagonists. The time-progressed degree or distance changes are shown for (LIG-D3.32 and S5.46-G7.52) LBS, (I3.40-F6.44, P5.50-F6.44 and I3.40 χ_1) P-I-F, (S3.39-N7.49 and C3.44-Y7.53) NPxxY, (R3.50-E6.30) ionic lock, and (R3.50-R6.35) TMH3-TMH6 distance.

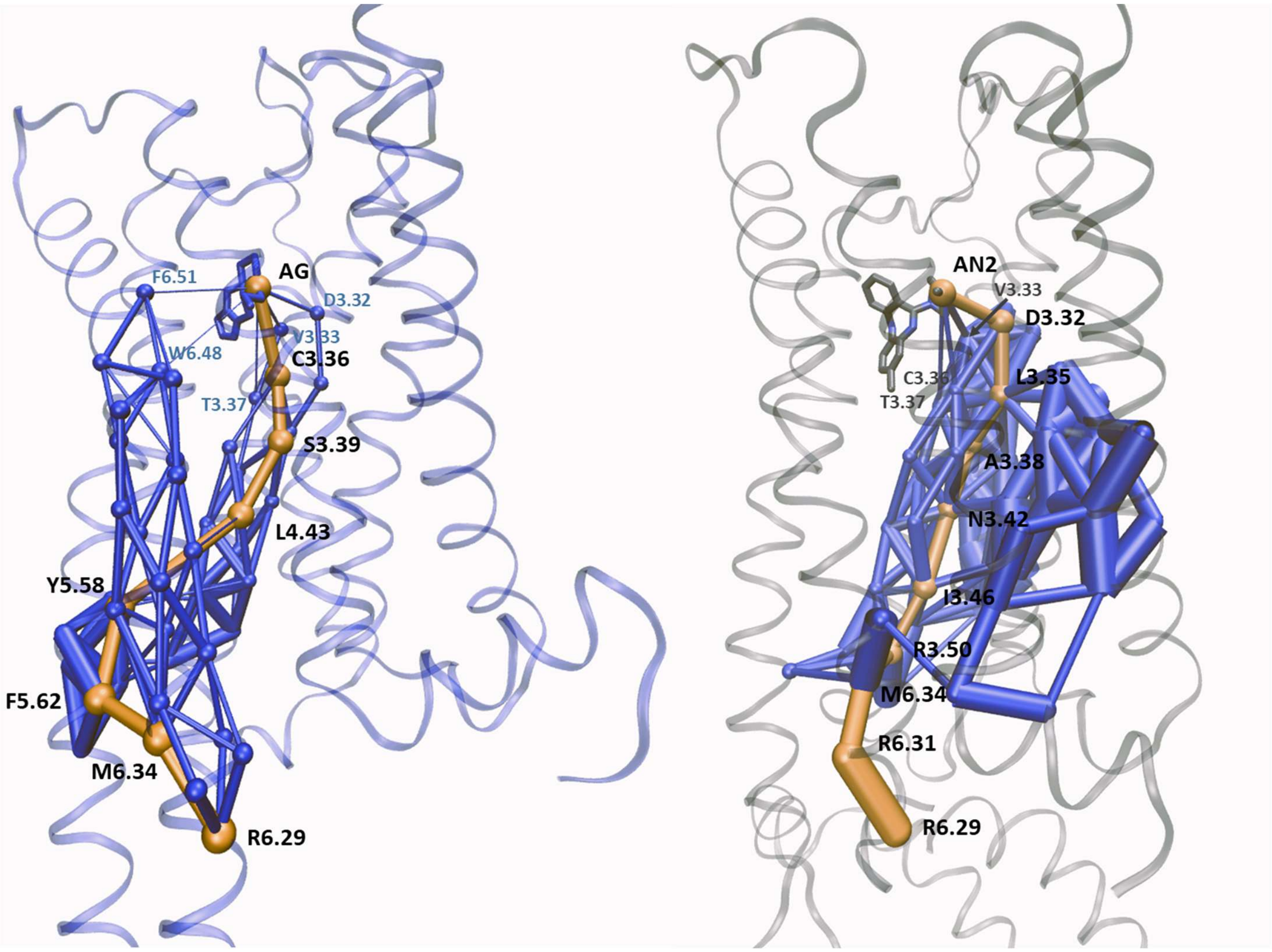

Figure S8. Visualisations of Dynamical Network Analysis of the dopamine D4 receptor complexes with example agonist (**AG2**) and antagonist (**AN2**). The active protein is shown as a purple ribbon, while the inactive is grey. The edges connecting nodes, i.e. communicating residues, are shown as blue lines, where thickness represents their weights, which can be interpreted as their communication strength. Orange-coloured suboptimal paths between ligand and R6.29 at the most intracellular end of TM6 are outlined. The initial nodes for the alternative paths are indicated in smaller font sizes.

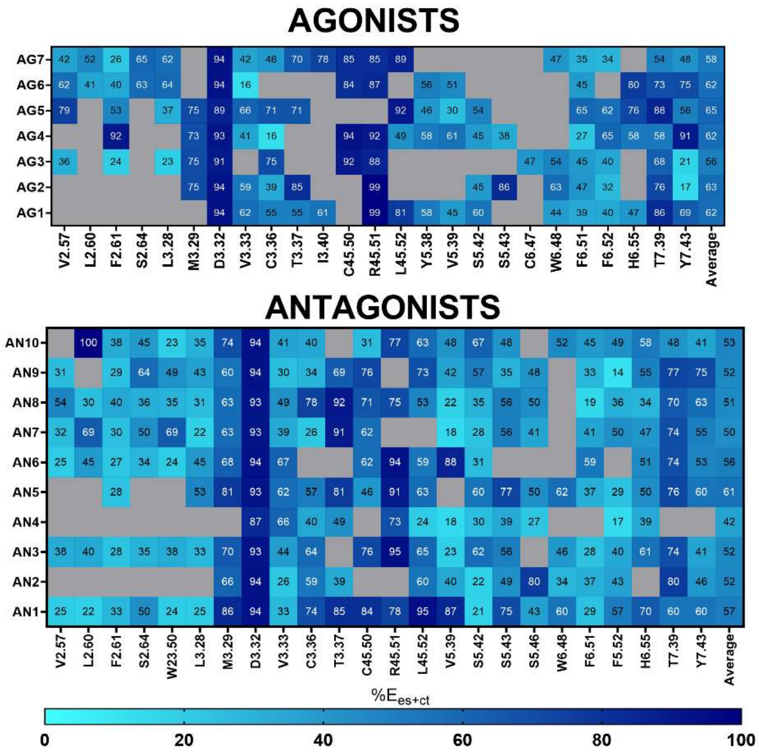

Figure S9. Comparison of PIEDA contribution in specific ligand–receptor interactions. In the heat map, if the contact is present, boxes are coloured according to their $\%E_{es+ct}$: from cyan (0% of sum electrostatics +charge-transfer and at the same time 100% of dispersion contributions) to dark blue (100% of sum electrostatics + charge-transfer).

Table S1. The free energy of the binding (ΔG_{bind}, kcal/mol) calculated with the molecular mechanics combined with the Poisson–Boltzmann and surface area continuum solvation method (MM/PBSA, [10.1517/17460441.2015.1032936]) based on the 100ns MD simulation and the interaction energy (E_{bind}, kcal/mol) calculated in the framework of the fragment molecular orbital methodology (FMO, [https://doi.org/10.1016/j.sbi.2021.08.010 for representative conformations of the human dopamine D4 receptor complexed with agonists (AG1-AG7) or antagonists (AN1-AN10).

	ΔG_{bind}	E_{bind}
AG1	-12.7	-148.5
AG2	-12.2	-149.2
AG3	-9.2	-163.1
AG4	-4.0	-115.9
AG5	-6.6	-166.2
AG6	-3.6	-155.9
AG7	-14.5	-183.8
AN1	-7.0	-177.5
AN2	-16.8	-179.4
AN3	-14.5	-173.8
AN4	-10.0	-49.9
AN5	-8.9	-171.4
AN6	-7.9	-159.2
AN7	-3.6	-201.7
AN8	-9.5	-209.5
AN9	-5.2	-184.8
AN10	-9.1	-176.6

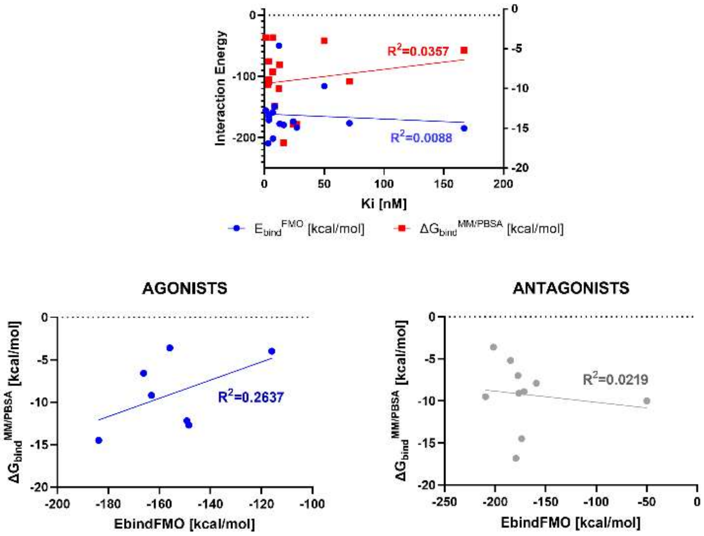

Supplement: Supplementary file 1 [file ijms-25-00746-s001.zip › ijms-2805393-supplementary.pdf]
